# ABO Gene Polymorphism and Thrombomodulin −33G>A Polymorphism Were Not Risk Factors for Myocardial Infarction in Javanese Men

**DOI:** 10.1155/2017/2943467

**Published:** 2017-07-24

**Authors:** Mifetika Lukitasari, Ahmad Hamim Sadewa, Mohammad Saifur Rohman

**Affiliations:** ^1^Department of Nursing, Faculty of Medicine, Brawijaya University, Malang, Indonesia; ^2^Faculty of Medicine, Gadjah Mada University, Yogyakarta, Indonesia; ^3^Department of Cardiology and Vascular Medicine, Faculty of Medicine, Brawijaya University, Malang, Indonesia

## Abstract

Genetic factors contribute to about a half of coronary artery diseases. During the last several decades, some studies suggested that non-O blood group and thrombomodulin polymorphism −33G>A are the risk factors of coronary artery disease especially in Asia. There was no prior study in Indonesia regarding this issue. Hence, this study was designed to investigate the correlation of ABO polymorphism and thrombomodulin polymorphism −33G>A with the incidence of acute myocardial infarction (AMI). A total of 192 subjects were enrolled in this case control study. AMI patients were diagnosed based on World Health Organization criteria. Healthy patients were subjects with AMI risk factor without any sign and symptoms of AMI. Patients with diabetes mellitus, cancer, and arrhythmia were excluded from this study. Genotyping for both polymorphisms was performed by PCR RFLP methods. The result of this study suggested that ABO polymorphism and thrombomodulin polymorphism −33G>A were not risk factors of AMI, *p* = 0.727 and *p* = 0.699, respectively. Furthermore, the analysis to identify the synergy of these polymorphisms failed to prove their correlation with AMI (*p* = 0.118). Conclusively, this study showed that ABO polymorphism and thrombomodulin polymorphism −33G>A were not risk factors of AMI.

## 1. Introduction

Coronary artery disease is the leading cause of death and disability in the world [1]. Acute myocardial infarction (AMI) is the most serious clinical manifestation of coronary artery disease leading to irreversible heart necrosis due to prolonged ischemia [2]. Genetic factors contribute to about a half of coronary artery diseases [3].

During the last several decades, some studies suggested that non-O blood group was a risk factor of myocardial infarction. Previous study on Caucasians revealed that blood group was a determinant of myocardial infarction susceptibility [4]. There is no prior study in Indonesia regarding this issue. This issue was explained by modulation of von-Willebrand Factor (vWF) by A and B glycosyltransferase [5]. Moreover, non-O blood group has 25% higher level of vWF compared to that of O blood group [6].

Thrombomodulin polymorphism −33G>A was reported as a determinant of myocardial infarction susceptibility in Asia [7–9]. Thrombomodulin gene produces thrombomodulin as a receptor in endothelial cell surface area and makes a complex with thrombin. This complex activates C protein that cleaves factor V and factor VIII. Thrombin-thrombomodulin complex will lose all of its procoagulant activity [10]. Polymorphism in the promoter region of this gene will lead to the decline of thrombomodulin gene expression [8, 11].

In Indonesia, we have no data regarding those issues; ethnic differences may contribute to this fact. Hence, this study was designed to investigate the correlation of ABO polymorphism and thrombomodulin polymorphism −33G>A with the incidence of acute myocardial infarction in Javanese men.

## 2. Patients and Method

### 2.1. Patients

A case control study was conducted in a total of one hundred and ninety-two subjects. Myocardial infarction was diagnosed by cardiologists based on World Health Organization criteria, that is, the manifestation of any 2 of the following 3 criteria: cardiac chest pain, electrocardiography changes in 2 consecutive leads that showed ST elevation or ST depression, and troponin I level increase in 4 hours after onset. Moreover, control subjects were patients with myocardial infarction risk factors such as smoking, hypertension, and dyslipidemia. Mostly, the control group was obtained from hypertensive community patients. Patients with diabetes mellitus, vascular disease, cancer, and arrhythmia were excluded from this study. This study excluded patients with diabetes mellitus and vascular disease since ABO gene and thrombomodulin −33G>A polymorphism were polymorphism in thrombotic pathway and diabetes mellitus and vascular disease significantly enhanced the thrombotic event that consequently enhances the acute myocardial infarction risk.

### 2.2. Methods

Thrombomodulin −33G>A polymorphism was identified by PCR RFLP method. The primers were forward: 5′-GGCCAGGGCTCGAGTTTATAAAGGC-3′ and reverse: 5′-CGGGGACAGTCGTCTTGTTACAGG-3′. Furthermore, the 259 bp PCR product was digested by Stu I enzyme and electrophoresed in 3% agarose gel. GG genotype resulted in 235 bp, AG genotype 235 bp and 24 bp, and AA genotype 235 bp [10].

ABO polymorphism was identified by PCR RFLP method with the primers shown in Table 1. Furthermore, the PCR product was digested by Kpn and Alu I enzyme and electrophoresed in 3% agarose gel. The RFLP result was interpreted based on the previous research [12].

### 2.3. Statistical Analysis

The data were analyzed by chi-square test. Difference was found to be significant if *p* < 0.05.

### 2.4. Ethical Considerations

The study obtained ethical clearance from Medical and Health Research Ethics Committee (MHREC), Faculty of Medicine, Gadjah Mada University, Dr. Sardjito General Hospital. The informed consent was obtained prior to study enrollment.

## 3. Results

The baseline characteristics of the sample (Table 2) showed that there were no significant differences in age, body height, and the presence of dyslipidemia. Significant difference between two groups was observed in body mass index, smoking status, and hypertension. The incidence of hypertension was significant in control group since most of the control group was obtained from hypertensive community patients.

Chi-square analysis of ABO polymorphism based on O and non-O blood group with the incidence of myocardial infarction showed that both blood groups had similar risk factor of myocardial infarction (Table 3). Furthermore, analysis between thrombomodulin −33G>A polymorphism and myocardial infarction suggested that carriers of GG genotype had similar risk factor of myocardial infarction compared to that of AG/AA genotype (Table 4). Moreover, analysis of the synergistic effect of both polymorphisms on myocardial infarction showed no significant difference. All groups showed no difference in myocardial infarction susceptibility compared to that of the reference gene (Table 5).

## 4. Discussion

This study revealed different proportion of ABO phenotype compared to those of other populations. A study in Pakistan showed that the prevalence of blood group in ischemic heart disease was A 34%, B 29%, AB 14%, and O 23% [13]. This study showed the prevalence of blood group phenotype among myocardial infarction patients was A 54.5%, B 21.2%, O 22.2%, and AB 2%.

Some previous studies on Caucasians suggested the correlation of ABO polymorphism and myocardial infarction [14–17]. This study categorized the sample based on O and non-O blood group. The result of this study is similar to previous study in China and South China (Table 6). Both studies used more than 2000 samples and identified some gene polymorphisms that became the risk factor of myocardial infarction in Caucasians in China population. Both studies revealed that ABO polymorphism that increases the susceptibility of myocardial infarction in Caucasian population failed to give significant result in China population [18, 19]. Ethnicity difference may contribute to the result of this study.

ABO polymorphism increases the susceptibility to myocardial infarction through some pathways, such as increasing vWF level, soluble intercellular adhesion molecule-1 level, soluble P-selectin level, and soluble E-selectin level [20]. However, the half-life of vWF and other cytokines was short (around 10 hours); therefore, we could not measure the vWF level. Moreover, ABO polymorphism affected circulated lipoprotein, triglyceride, and total cholesterol level [21]. This study revealed no significant difference in total cholesterol, triglyceride, HDL, and LDL between O and non-O blood group. This fact might explain the result of this study.

ABO polymorphism cannot affect the myocardial infarction susceptibility solely. There must be another gene that synergistically increases the susceptibility. Thrombomodulin gene polymorphism was a polymorphism that is more commonly found in Asian population. However, some studies in other Asia regions suggested conflicting result (Table 7). A study in Korea revealed that carriers of −33G>A thrombomodulin polymorphism had 4.63 times higher susceptibility to myocardial infarction [8]. Moreover, meta-analysis by Wang and Dong suggested that −33G>A polymorphism increases the susceptibility to coronary artery disease (OR = 1,65; 95% CI, 1,35–2,02; *p* < 0,01; *I*^2^  = 15%) [22]. However, this study revealed no significant correlation between thrombomodulin polymorphism −33G>A and myocardial infarction. A study by Zhao et al. in 808 coronary artery disease patients and 813 healthy patients also failed to demonstrate a significant result [10]. Different genetic variation in different population might explain these different results.

## 5. Conclusion

This study showed that ABO polymorphism and thrombomodulin polymorphism −33G>A were not risk factors of AMI in Javanese males.

## Figures and Tables

**Table 1:** Primer in ABO polymorphism identification.

Primer	Sequence (5′-3′)	Name
ABO-1	GCAGTAGGAAGGATGTCCTC	A7500 (261F)
ABO-2	AATGTCCACAGTCACTCGCC	A7501 (261R)
ABO-3	TGGAGATCCTGACTCCGCTG	A7502 (703F)
ABO-4	GTAGAAATCGCCCTCGTCCTT	A7503 (703R)

**Table 2:** Baseline characteristics.

Variables	Case (*n* = 99)	Control (*n* = 93)	*p*
Age	56,45 ± 9,70	56,19 ± 9,79	0,853^*∗*^
BMI (kg/m^2^)			
Normal (18.25 to <25)	39 (39,4)	23 (24,7)	0,004^#^
Overweight (25 to <30)	24 (24,2)	18 (19,4)	
Obese 1 (30 to <35)	26 (26,3)	42 (45,2)	
Obese 2 (35 to <40)	6 (6,1)	10 (10,8)	
Dyslipidemia (yes)	39 (39,4)	40 (43)	0,717^#^
Smoking (yes)	81 (81,8)	42 (45,2)	0,000^#^
HT (yes)	42 (42,4)	83 (89,2)	0,000^#^

Data is represented in *n* (%). ^*∗*^Data were analyzed by independent *t*-test. ^#^Data were analyzed by chi-square test. BMI: body mass index.

**Table 3:** Association between ABO gene polymorphism −33G>A and acute myocardial infarct based on blood groups O and non-O.

Variables	Cases (*n* = 99)	Control (*n* = 93)	*p*	OR	95% CI
O	22 (22,2)	17 (18,3)	0,618	0,727	0,355–1,490
Non-O	77 (77,8)	76 (81,7)			

Data presented in *n* (%).

**Table 4:** Association of thrombomodulin gene polymorphism −33G>A with acute myocardial infarction.

Variables	Case (*n* = 99)	Control (*n* = 93)	*p*	OR	95% CI
GG	91 (91,9)	83 (89,2)	0,699	1,37	0,516–3,637
AG/AA	8 (8,1)	10 (10,8)			

Data presented in *n* (%).

**Table 5:** Synergistic association between thrombomodulin −33G>A gene polymorphisms and ABO gene polymorphism with the occurrence of acute myocardial infarct.

Variables	Case (*n* = 99)	Control (*n* = 93)	*p*	OR	95% CI
GG/O	22 (22,2)	14 (15,1)	Reference gene		
GG/non-O	71 (71,8)	69 (74,2)	0,354	0,5	0,423–0,591
AG/AA and non-O	6 (6)	7 (7,5)	0,544	1,833	0,51–6,593
AG/AA and O	0 (0)	3 (3,2)	0,148	0,389	0,258–0,586

Data presented in *n* (%).

**Table 6:** Research summary of ABO gene polymorphisms on other populations.

Author	Research subject	Sample number	OR (95% CI)
Wang et al.	China subject		1,03 (0,8–1,33)
	AMI cases	2365	
	Control	2768	

Huang et al.	Southern China subject		1,279 (0,713–2,452)
	AMI cases	1716	
	Control	1572	

Biswas et al.	Indian subject		1,857 (1,112–3.1)
	CAD cases	250	
	Control	250	

AMI: acute myocardial infarction and CAD: coronary acute disease.

**Table 7:** Research summary of thrombomodulin gene polymorphisms on other populations.

Author	Research subject	Genotype			OR (95% CI)
		GG	AG/AA	Total	
Park et al.	Korean subject				
	AMI 1-VD	18	12	30	4,36 (1,62–13,31)
	Control	86	16	102	

Li et al.	China and Taiwan subject				
	AMI cases	215	63	278	1,6 (1,1–2,5)
	Premature AMI cases	51	21	72	2,3 (1,3–4,1)
	Control	377	73	450	

Li et al.	China and Taiwan subject				
	CAD cases	169	31	200	1,81 (1,11–2,92)
	Control	244	76	320	

Zhao et al.	China and North Han				
	AMI cases	644	164	808	1,25 (0,93–1,69)
	CAD cases	398	107	505	1,23 (0,88–1,73)
	Premature AMI	146	42	146	1,45 (0,87–2,44)
	Control	662	151	813	

Dogra et al.	North Indian				
	subject				
	AMI cases	182	7	189	0,5 (0,1–2,6)
	Control	343	2	345	

1-VD: one vessel disease; AMI: acute myocardial infarction; CAD: coronary acute disease.
